# Performance of crossbred pigs with indigenous and Hampshire inheritance under a smallholder production system in the Eastern Himalayan hill region

**DOI:** 10.3389/fgene.2023.1042554

**Published:** 2023-04-03

**Authors:** Govindasamy Kadirvel, Yumkhaibam Sovarani Devi, Syamal Naskar, K. M. Bujarbaruah, Gutum Khargariah, Santanu Banik, Ningthoujam Suraj Singh, Chamniugongliu Gonmei

**Affiliations:** ^1^ Division of Animal and Fisheries Sciences, ICAR Research Complex for NEH Region, Umiam, Meghalaya, India; ^2^ NDRI Regional Centre, Kolkata, India; ^3^ NRC on Pig, Guwahati, India

**Keywords:** crossbred pig, Niang Megha, Hampshire inheritance, performance, Eastern Himalayan, hill ecosystem

## Abstract

Pig productivity is very low in the Eastern Himalayan hill region due to the poor performance of local pigs. To improve pig productivity, it was decided to develop a crossbred pig of Niang Megha indigenous and Hampshire as an exotic germplasm. The performance of crossbred pigs with different levels of Hampshire and indigenous inheritance—H-50 × NM-50 (HN-50), H-75 × NM-25 (HN-75), and H-87.5 × NM-12.5 (HN-87.5)—was compared for their performance to find a suitable level of genetic inheritance. Among the crossbreds, HN-75 performed better in terms of production, reproduction performance, and adaptability. *Inter se* mating and selection were carried out on six generations of HN-75 pigs, and genetic gain and trait stability were evaluated and released as a crossbred. These crossbred pigs attained body weights of 77.5–90.7 kg by 10 months of age, with FCR of 4.3:1. Age at puberty was 276.66 ± 2.25 days, and average birth weight was 0.92 ± 0.06 kg. Litter size at birth and weaning were 9.12 ± 0.55 and 8.52 ± 0.81. These pigs have good mothering abilities with a weaning percentage of 89.32 ± 2.52%, good carcass quality, and consumer preference. The lifetime productivity for an average of six farrowings/sow showed a total litter size at birth of 51.83 ± 1.61 and total litter size at weaning of 47.17 ± 2.69. In a smallholder production system, the crossbred pigs showed a better growth rate and a higher litter size at birth and at weaning than average local pigs. Hence, the popularization of this crossbreed would enhance the production, productivity, livelihood, and income of the regionʼs farmers.

## 1 Introduction

The Eastern Himalayan hill region of India has a distinct ecosystem, topography, and biodiversity. This subtropical hill region has less than 15% cultivable land; almost 90% of the area is covered by evergreen forest (Poffenberger et al., 2007) and mostly inhabited by tribal ethnicities (Govt. of India, 2011). Livestock plays a crucial role in the nutritional security, income, and livelihood of the farmers in the region. Among the various forms of livestock, pigs are the most popular and valued species and are an integral part of the diversified resource-poor agriculture in the region, especially among the tribal communities (Rangnekar, 2006; Banik et al., 2013; Jain, 2016). Pigs have a special significance in the socio-economic status of the farmers (Kadirvel et al., 2013). Pork is the most preferred meat among the population; this region has much higher pork consumption than the rest of the country (Kumaresan et al., 2006a). Due to the importance of pig in this region's dietary habits, almost every rural household rears two to three pigs as a livelihood resource (Kadirvel et al., 2013). However, pigs are reared under a smallholder low-input production system which utilizes locally available resources like agricultural bio-products and kitchen wastes as they feed off less than 1 ha of land (Kumaresan et al., 2007; Haldar et al., 2017; Kadirvel et al., 2017). The total pig population of India is 9.06 million, of which 7.16 million (79.03%) are contributed by indigenous and local pigs (Livestock census, 2019). The north-eastern states of India constitute almost half the of country's total pig population: 46.80% (Basic Animal Husbandry Fisheries Statistics, 2019). Low quality local pigs comprise 67.90% of the region's total pig population. Although a considerable pig population is present there, the productivity of the pigs is low due to the poor productive and reproductive performance of local pigs (Kadirvel et al., 2021). In order to improve pig productivity in the region and the preference for crossbred pigs among local farmers, a project was developed to crossbreed pigs with the Niang Megha pig as indigenous germplasm for better adaptability and Hampshire as an exotic germplasm for enhanced productivity in the hill ecosystem of the North-Eastern Hill (NEH) region of India. The indigenous pig Niang Megha was selected for the study since they have evolved over many years and are well suited in the hilly, low-input traditional tribal production system (Rajbongshi et al., 2017). Hampshire has been used extensively for breeding purpose for up-grading local pigs as it has well-balanced productive and reproductive performance in tropical humid environments (Kumaresan et al., 2006b; Oke et al., 2006), as well as the preference for black coloured pigs among the farmers. The objective of this study was to develop a crossbred pig with indigenous and Hampshire inheritance. Planning for the development of the crossbred pig was initiated in 1998—a crossbreeding program with rigorous selection. Further study was conducted to evaluate the performance of different traits of economic importance (including both productivity and adaptability traits), which resulted in the development of a crossbred pig named “Lumsniang,” based on the locality and its features.

## 2 Materials and methods

### 2.1 Study location

Experiments I and II were conducted in the pig breeding farm of the ICAR Research Complex for the NEH Region. The farm is located at 24.58°N to 26.07°N latitude and 89.48°E to 92.51°E longitude with an altitude of 1,010 m above mean sea level. Annual minimum, maximum, and mean temperatures are 13.06°C, 25.46°C, and 19.26°C, respectively. Relative humidity varies from 65% to 81.70% with an average of 72.24%. Experiment III was conducted in a smallholder production system with farmers having experience of rearing low-quality pigs. The agro-climatic conditions were similar, and the experiments were conducted in the same region of nearby villages/cluster of the institute. The study site is located 1,005–1120 m above mean sea level, which is in a high rainfall area of 2,239–2,953 mm annually. A subtropical climate prevails in the study area, with annual maximum and minimum temperatures ranging from 21.1 to 29.2°C and 7.0 to 20.9°C, respectively. In the study area, pig husbandry plays a significant role in supporting the social, cultural, and economic livelihood of the tribal people in the location. The pigs are mostly reared under a traditional smallholder low-input production system where every tribal household rears two to three average-quality pigs in their backyard, as reported previously (Kadirvel et al., 2017). Rice and pork are the staple foods in the study location; hence, pork is in great demand as a meat.

### 2.2 Management system

The pigs in the study were reared under an intensive management system and housed according to their sex, age, and physiological condition. Pregnant sows were transferred to farrowing pens 1 month before farrowing. Mature boars were kept in individual pens. Piglets were brooded and fed commercial mesh *ad libitum* as per standard recommendation—pig starter feed containing 22% crude protein and 3300 ME/kg. Protein contents of 18% for weaned piglets up to 3 months of age, 15% for growers, 16% for breeding boars and pregnant sows, and 14% for finisher/dry sows were incorporated in the ration. Drinking water was provided *ad libitum* throughout the period. Piglets were weaned at 56 days old. Iron injections were given on the 4th and 14th days, deworming and vaccination were carried out regularly, and other therapeutic treatments were provided as needed. Mating was carried out through natural service.

### 2.3 Breeding management

**FIGURE 1:**
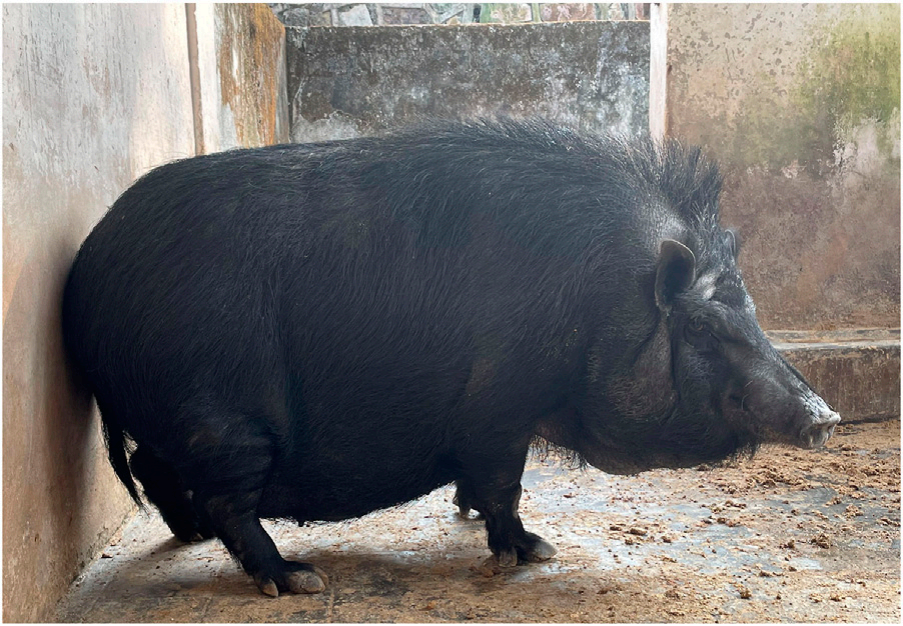
Indigenous breed: adult Niang Megha pig.

**FIGURE 2:**
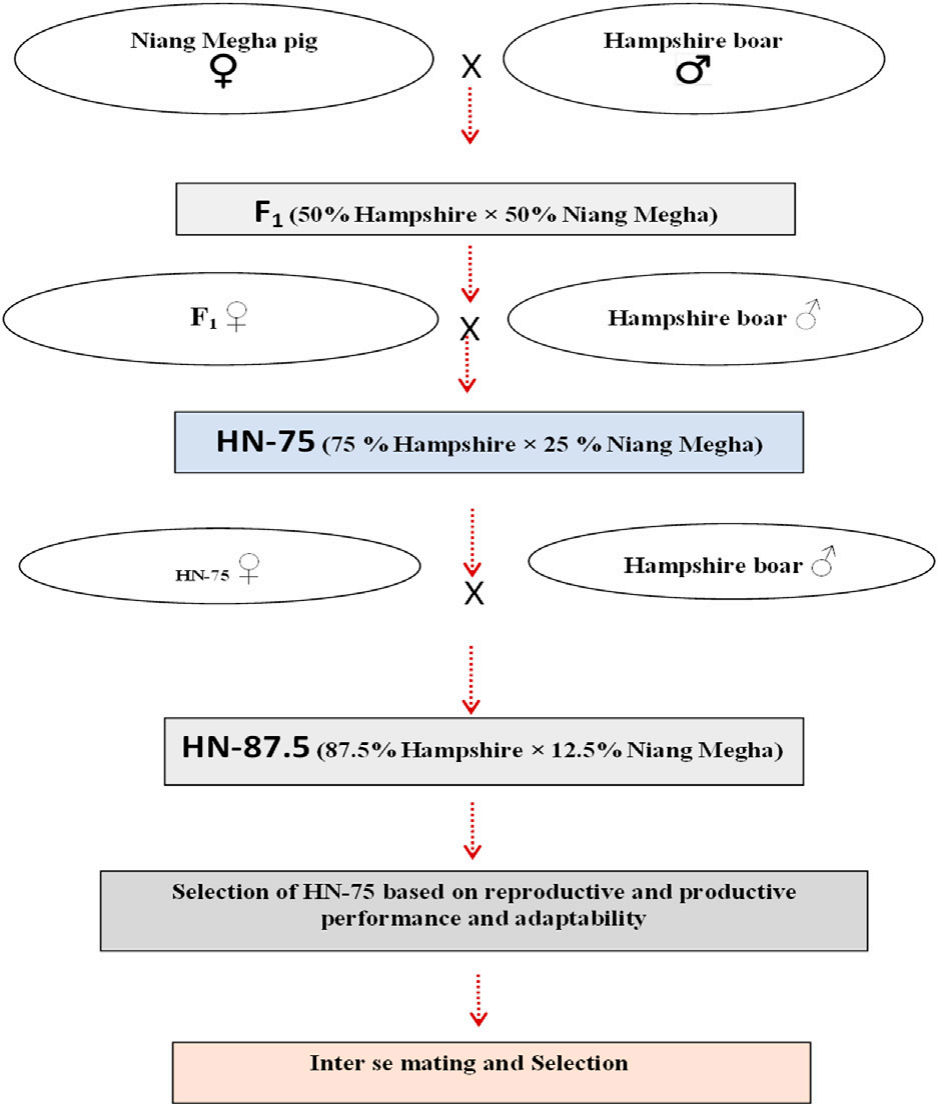
Crossbreeding program.

Niang Megha (NM), a registered small-sized indigenous breed of pig having a 35–40 kg body weight at 10 months of age, was used as the dam line (Figure 1). Some 40 pure NM were purchased in a sex ratio of 1:3 from different parts of its home tract to avoid inbreeding based on pedigree, phenotypic, and morphometric characteristics true to NM; they were maintained at the institute's pig breeding farm. These indigenous pigs have poor productive and reproductive performance. Similarly, 40 pure Hampshire pigs for the sire line were procured from pig breeding farm at Kyrdemkulai, Government of Meghalaya, and maintained under the same conditions. A group of selected NM gilts was bred with pure Hampshire boars to achieve 50% Hampshire-inheritance crossbred pigs. To select male and female F_1_, male animals were selected based on weaning weight and 8-month body weight, in a two-stage sequential selection. Female animals were selected on the dam’s litter size at birth (>7) and the weaning weight and number of functional teats (at least six pairs of functional teats). The progeny of F_1_ crossbred HN-50 (50% H × 50% NM) gilts were again backcrossed with Hampshire boars to produce crossbred HN-75 (75% H, 25% NM) pig. Pure-breed Hampshire boars were utilized to produce crossbred HN-87.5 (87.5% H and 12.5% NM). A 1:3 sex ratio of male to female animals was maintained to avoid inbreeding effects in the farm. The crossbred pigs with the desired level of exotic inheritance were maintained by *inter se* mating following strict selection for six generations for stabilization of (re)productive performance. The cross-breeding strategy followed in the present study is depicted in Figure 2.

#### 2.3.1 Experiment I: Comparative performance of crossbred pigs with different levels of genetic inheritance

The performance of NM and crossbred pigs with different levels of Hampshire inheritance was compared with respect to their productive and reproductive performance, as well as their incidence of disease. The study was conducted to identify a suitable level of exotic inheritance for adaptability and better performance in the hill ecosystem. Data for this comparative study were obtained from pigs of the four genetic groups—NM, HN-50, HN-75, and HN-87.5—spread over 7 years from 1998 to 2006. A random sample of 55 piglets from each genetic group was selected for productive performance. From each genetic group, 25 random sows were selected to study reproductive performance at puberty, first conception, inter-farrowing interval, and litter size at birth and at weaning. A total of 30 random adult pigs from each genetic group were slaughtered at 10 months old over the years to study carcass traits. During this period, the incidence of different diseases was also recorded for each genetic group. The medicine and veterinary cost per year was also calculated by dividing the total expenditure by the number of pigs in each genetic group. Based on the phenotypic performance, HN-75 was selected in 2006 for further improvement.

#### 2.3.2 Experiment II: *Inter se* mating and evaluation of selected crossbred pigs

Data were obtained from 240 pigs for production performance and from 30 breeding sows for reproduction traits over 9 years from 2006 to 2015. The selection of the sows was based on their lifetime productivity (over six generations) based on the number of piglets born over their lifetime, litter size at birth, weaning weight, litter weight at birth, and number of functional teats. Similarly, the selection of boars was based on phenotypic performance such as body conformity, presence of well-developed testicles, birth weight, weaning weight, and individual body weight as per age. The overall and generational genetic gain of the crossbred variety was estimated for different productive and reproductive parameters. After stable performance for 3–4 years in terms of productive and reproductive traits, the pigs were considered crossbred.

#### 2.3.3 Experiment III: Performance evaluation of crossbred pigs under a smallholder production system

To evaluate the crossbreed, farmers rearing average local pigs were selected from 20 villages; 100 units of the crossbred pig were established, each unit consisting of two female animals and one male animal under the smallholder production system. Data were obtained from a total of 120 piglets for growth performance and 50 sows for reproductive traits over 3 years. The pigs were maintained in the pen system of housing made of locally available materials. Pigsties were made of either concrete, wooden planks, or bamboo poles with a tin roof. These pigs were fed different levels of concentrate feed/feed ingredients purchased from market, in addition to local agro-wastes and household kitchen wastes. Training in modern pig husbandry management with continuous technological backup was provided to the farmers as well as the provision of healthcare management. These farmers were also advised to carry out regular deworming and vaccination and to perform improved management practices, including preparation of low-cost feed formulation with locally available feed resources. The performance of crossbred piglets was monitored at monthly intervals to record their health, growth rate, and reproductive parameters. To compare their performance with existing local pigs, the same numbers of the latter were selected from different households in the same clusters/villages. The pig units were visited by project staff and monitored monthly to record their health, incidence of diseases conditions, growth rate, and reproductive parameters. These farmers were also advised to carry out regular deworming and vaccination and to follow improved management practices. Market demand as well as consumer preference for crossbred or average pigs was assessed using a pretested survey format. The 100 individual farmers interviewed on the market demand and consumer preference were scored using a scale from 1 (poor) to 5 (excellent).

### 2.4 Statistical analysis

All the collected data were analysed using SPSS statistical software 2008 (SPSS, 2008). Multiple ANOVA was performed to check if the means of various traits and market demand among the genetic groups were different at the 5% level of significance (*p* ≤ 0.05). For disease incidence, the Kruskal–Wallis H test was conducted with binary data for significant difference among the genetic groups. Duncan’s multiple range test was performed to make all pairwise comparisons among the means of traits of different genetic groups wherever a significant difference was obtained.

## 3 Results

### 3.1 Experiment I: Comparative performance of crossbred pigs with different levels of genetic inheritance

The productive performance of NM, HN-50, HN-75, and HN-87.5 is presented in Table 1. Pre- and post-weaning growth rates were significantly higher in the crossbred pigs than NM. Among the crossbreds, HN-87.5 had a significantly higher pre-weaning (153.57 ± 1.71 g/day) and post-weaning growth rate (332.17 ± 1.27 g/day) than HN-50 and HN-75. Body weight at all age groups was significantly higher in HN-87.5 than other genetic groups. HN-87.5 pigs attained the highest body weight of 89.54 ± 0.97 kg at 10 months old, followed by HN-75 (83.92 ± 0.67 kg), HN-50 (65.21 ± 0.98 kg), and NM pigs (37.63 ± 0.86 kg).

**TABLE 1:** Comparison of productive traits of different genetic groups of pigs (Mean ± S.E.).

Parameter	Niang Megha (50)	HN-50 (50)	HN-75 (50)	HN-87.5 (50)
Pre-weaning growth rate (g/d)	84.45^a^ ± 1.21	106.45^b^ ± 1.29	133.45^c^ ± 1.34	153.57^d^ ± 1.71
Post-weaning growth rate (g/d)	133.55^a^ ± 1.91	240.87^b^ ± 0.84	320.55^c^ ± 1.34	332.17^d^ ± 1.27
Body weight at different ages (kg)				
60 days	5.59^a^ ± 0.03	7.40^b^ ± 0.44	9.00^c^ ± 0.36	10.54^d^ ± 0.47
120 days	11.55^a^ ± 0.52	16.68^b^ ± 0.37	19.22^c^ ± 0.52	24.63^d^ ± 0.57
180 days	19.97^a^ ± 0.42	29.35^b^ ± 0.76	42.56^c^ ± 0.79	47.02^d^ ± 0.69
240 days	28.75^a^ ± 0.76	42.53^b^ ± 0.83	65.87^c^ ± 0.67	68.72^d^ ± 0.75
300 days	37.63^a^ ± 0.86	65.21^b^ ± 0.98	83.92^c^ ± 0.77	89.54^d^ ± 0.97

^a-b^ values in the same row with different superscript differ significantly (*p* ≤ 0.05). Means with different superscripts in respective rows differ significantly (*p* ≤ 0.05). Figures in parenthesis indicate the number of observations. NM: Niang Megha; HN-50: 50% H × 50% NM; HN-75: 75% H × 25% NM; HN-87.5: 87.5% H × 12.5% NM.

Age at puberty, age at first farrowing, and inter-farrowing interval increased in the crossbred pigs with increased exotic inheritance (Table 2). Litter size at birth was significantly higher (*p* ≤ 0.05) in HN-87.5 compared to other genetic groups; however, no significant difference was observed for litter size at weaning. Hence, HN-87.5 was found to have a significantly lower weaning percentage than other genetic groups, mainly due to crushing, which indicates poor mothering ability. HN-75 was found to have better litter performance than HN-50, although there was no significant difference. However, traits such as age at puberty, at first conception, and at first farrowing were significantly earlier in NM than in crossbred pigs due to earlier sexual maturity.

**TABLE 2:** Comparison of various reproductive traits of different genetic groups of pigs (Mean ± S.E.).

Trait	NM (Tribout et al., 2010)	HN-50 (Tribout et al., 2010)	HN-75 (Tribout et al., 2010)	HN-87.5 (Tribout et al., 2010)
Age at puberty (days)	213.19^a^ ± 2.86	266.38^b^ ± 1.19	293.16^bc^±1.19	306.31^cd^ ± 1.45
Age at first conception (days)	240.35^a^ ± 1.92	300.15^b^ ± 2.41	331.13^bc^±1.41	346.32^cd^ ± 1.35
Age at first farrowing (days)	362.67^a^ ± 2.95	424.25^b^ ± 2.52	432.17^bc^±1.52	478.50^cd^ ± 2.50
Inter-farrowing interval (days)	210.33^a^ ± 1.42	215.46^a^ ± 1.16	208.04^a^ ± 2.16	220.50^b^ ± 1.75
Litter size at birth (no.)	5.80^a^ ± 0.42	7.52^b^ ± 0.85	8.72^b^ ± 0.75	9.28^c^ ± 0.33
Litter size at weaning (no.)	4.57^a^ ± 0.48	7.11^b^ ± 1.81	8.05^b^ ± 0.52	8.42^b^ ± 0.73
Birth weight (kg)	0.54^a^ ± 0.34	0.79^b^ ± 0.16	0.83^b^ ± 0.16	0.94^b^ ± 0.21
Weaning weight (kg)	5.25^a^ ± 0.44	6.78^b^ ± 0.14	8.32^b^ ± 1.14	9.54^b^ ± 0.47
Av. weaning percentage (%)	80.35^a^ ± 0.77	92.82^b^ ± 0.72	87.36^c^ ± 0.75	85.28^d^ ± 0.76

^a-b^ values in the same row with different superscript differ significantly (*p* ≤ 0.05). Means with different superscripts in respective rows differ significantly (*p* ≤ 0.05). Figures in parenthesis indicate number of observations. NM: Niang Megha; HN-50: 50% H × 50% NM; HN-75: 75%H × 25% NM; HN-87.5: 87.5% H × 12.5% NM.

The incidence of different diseases as well as mortality patterns varies with different genetic groups of pigs (Table 3). The incidence of stillbirth and crushing of piglets was found to be significantly (*p* ≤ 0.05) higher in HN-87.5 pigs than other genetic groups. Similarly, piglet diarrhoea was found to be significantly (*p* ≤ 0.05) higher in HN-87.5 (10.23 ± 0.33%), followed by HN-75 (8.53 ± 0.31%) and HN-50 (8.24 ± 0.23%) and was lowest in NM (6.78 ± 0.11%). Pre-weaning mortality was found to be significantly higher (*p* ≤ 0.05) in HN-87.5 pigs (8.43 ± 0.32%) than other genetic groups. Post-weaning mortality was significantly higher (*p* ≤ 0.05) in HN-87.5 than in HN-50, but there was no significant difference with HN-75. However, adult mortality was significantly higher (*p* ≤ 0.05) in HN-87.5 than for other genetic groups. The medicine and veterinary costs per year were also highest in HN-87.5 crossbred pigs (Table 3).

**TABLE 3:** Incidence (%) of major disease conditions in different genetic groups of pigs (mean ± S.E.).

Diseases condition	NM	HN-50	HN-75	HN-87.5
Pre-weaning				
Stillbirth	0.23^a^ ± 0.00	0.37^a^ ± 0.00	0.73^b^ ± 0.01	0.88^b^ ± 0.02
Crushing of piglets	0.72^a^ ± 0.00	0.15^b^ ± 0.00	1.20^c^ ± 0.03	2.67^d^ ± 0.07
Weak piglets	0.07^a^ ± 0.00	0.03^b^ ± 0.00	0.07^a^ ± 0.00	0.12^c^ ± 0.00
Piglet diarrhoea	6.78^a^ ± 0.11	8.24^b^ ± 0.23	8.53^b^ ± 0.31	10.23^c^ ± 0.33
Mortality (%)	6.76^a^ ± 0.21	5.34^a^ ± 0.15	6.13^a^ ± 0.21	8.43^b^ ± 0.32
Post-weaning				
Piglet diarrhoea	3.41^a^ ± 0.07	3.72^a^ ± 0.08	4.23^b^ ± 0.11	4.87^b^ ± 0.13
Wound/abscess/ear bit/leg-lesion/other body lesions	9.73^a^ ± 0.03	12.31^a^ ± 0.02	16.72^b^ ± 0.31	21.34^c^ ± 0.37
Pneumonia	4.52^a^ ± 0.03	3.73^a^ ± 0.02	4.34^a^ ± 0.03	4.57^a^ ± 0.02
Skin diseases/lesions	5.72^a^ ± 0.02	5.78^a^ ± 0.03	7.45^b^ ± 0.04	12.4^c^ ± 0.11
Weakness	2.31^a^ ± 0.01	3.52^b^ ± 0.02	3.78^b^ ± 0.12	5.3^c^ ± 0.91
Lameness/arthritis/hoof lesions	1.22^a^ ± 0.01	1.87^a^ ± 0.02	3.52^b^ ± 0.11	5.72^c^ ± 0.12
Metritis	0.23^a^ ± 0.00	0.34^a^ ± 0.00	0.78^b^ ± 0.00	0.84^b^ ± 0.00
Other minor (uterine prolapse)	1.23^a^ ± 0.01	1.45^a^ ± 0.01	3.6^b^ ± 0.02	4.87^c^ ± 0.4
Mortality %	2.01^a^ ± 0.01	2.13^a^ ± 0.01	3.42^b^ ± 0.02	3.76^b^ ± 0.02
Adult mortality %	0.44^a^ ± 0.00	0.76^a^ ± 0.00	1.21^b^ ± 0.01	1.76^c^ ± 0.01
Medicine and veterinary costs/year (INR)	387.55^a^ ± 2.32	427.21^a^ ± 2.50	467.35^b^ ± 3.71	523.22^c^ ± 3.91

^a-b^ values in the same row with different superscript differ significantly (*p* ≤ 0.05). Means with different superscripts in respective rows differ significantly (*p* ≤ 0.05). NM: Niang Megha; HN-50: 50% H × 50% NM; HN-75: 75% H × 25% NM; HN-87.5: 87.5% H × 12.5% NM.

It was noted from this comparative study that HN-87.5 has better productive performance than the other genetic groups; however, an exotic inheritance level that exceeds 75% can result in a longer inter-farrowing interval, poorer weaning percentage due to poorer mothering ability, higher incidence of different disease conditions, and higher mortality. Crossbred HN-75 had overall better phenotypic performance and better adaptability in terms of disease resistance. Therefore, this study selected crossbred pigs with 75% Hampshire inheritance (HN-75) for further improvement.

### 3.2 Experiment II: *Inter se* mating and evaluation of selected crossbred pigs

Selected HN-75 pigs were maintained by *inter se* mating and important economic traits were evaluated for their stability, along with their performance over six generations. All parameters showed gradual improvement along the generations due to selection (Table 4). The overall genetic gains for litter size at birth and at weaning were 4.59 and 5.84%, respectively. Similarly, the genetic gain for birth and weaning weight were 10.84 and 13.70%, respectively, over the six generations. Body weight at 120 days was found to have the highest overall genetic gain (22.94%) among all the parameters considered.

**TABLE 4:** Improvement of performance and genetic gain of HN-75 crossbred pigs over six generations through selection (Mean ± S.E.).

Parameter	First generation	Second generation	Third generation	Fourth generation	Fifth generation	Sixth generation	Overall genetic gain (%)	Average genetic gain/generation (%)
Litter size at birth (no.)	8.72 ± 0.75	8.79 ± 0.87	8.81 ± 1.04	8.98 ± 0.17	9.02 ± 1.21	9.12 ± 0.55	4.59	0.76
Litter size at weaning (no.)	8.05 ± 0.52	8.12 ± 0.87	8.25 ± 0.25	8.27 ± 0.73	8.37 ± 0.21	8.52 ± 0.81	5.84	0.97
Body weight at (kg)								
Birth	0.83 ± 0.16	0.85 ± 0.25	0.87 ± 0.26	0.88 ± 0.16	0.89 ± 0.16	0.92 ± 0.06	10.84	1.81
Weaning	8.32 ± 0.34	8.63 ± 0.47	8.87 ± 0.48	9.05 ± 0.54	9.21 ± 0.87	9.46 ± 0.81	13.70	2.28
120 days	19.22 ± 0.52	19.98 ± 0.38	20.33 ± 0.49	20.43 ± 0.39	21.43 ± 0.58	23.63 ± 0.55	22.94	3.82
180 days	42.56 ± 0.79	43.54 ± 0.59	44.87 ± 0.62	45.16 ± 0.66	46.57 ± 0.57	47.13 ± 0.64	10.74	1.79
240 days	65.87 ± 0.67	65.69 ± 0.73	66.59 ± 0.58	67.20 ± 0.82	67.45 ± 0.78	68.11 ± 0.80	3.40	0.57
300 days	83.92 ± 0.67	83.79 ± 0.94	84.47 ± 0.87	85.21 ± 0.59	85.36 ± 0.67	86.48 ± 0.92	3.05	0.51

No. of observations is 240 for production traits and 30 for reproduction traits.

### 3.3 Performance of the crossbred pig selected after *inter se* mating

The performance of the crossbred pigs in terms of production, reproduction, and carcass traits was evaluated and the results are presented in Table 5. The average pre- and post-weaning growth rates were 143.50 ± 1.22 and 320.33 ± 1.55 g/day, respectively. The pigs attained the average body weight of 86.48 ± 0.92 kg at 10 months with ranges from 77.5 to 90.7 kg. Age at first conception was found to be 331.13 ± 2.65 days (Table 5). Litter size at birth was 9.12 ± 0.55 and at weaning was 8.52 ± 0.81. The crossbred pig variety was slaughtered at 10 months old to study the carcass traits. The average dressing percentage was 73.33 ± 0.37% with back-fat thickness of 2.30 ± 0.21 cm (Table 5). Lifetime productivity of the crossbred pig was also evaluated for six farrowings (Table 6). The crossbred pigs were found to have a total litter size at birth of 51.83 ± 1.61, whereas total litter size at weaning was 47.17 ± 2.69 in the present study

**TABLE 5:** Performance of crossbred pig variety.

Parameter	Mean ± SE
Production Performance (N = 85)	
1. Pre-weaning growth rate (g/d)	143.50 ± 1.22
2. Post-weaning growth rate (g/d)	320.33 ± 1.55
3. Feed conversion efficiency ()	1:4.30
4. Body weight at 120 days (kg)	23.63 ± 0.55
5. Body weight at 180 days (kg)	47.13 ± 0.64
6. Body weight at 240 days (kg)	68.11 ± 0.80
7. Body weight at 300 days (kg)	86.48 ± 0.92
Reproduction performance (N = 50)	
1. Age at puberty (days)	276.66 ± 2.23
2. Age at first conception (days)	331.13 ± 2.65
3. Age at first farrowing (days)	425.26 ± 2.82
4. Inter-farrowing intervals (days)	205.04 ± 1.82
5. Litter size at birth (no.)	9.12 ± 0.55
6. Litter size at weaning (no.)	8.52 ± 0.81
7. Birth weight (kg)	0.92 ± 0.06
8. Weaning weight (kg)	9.46 ± 0.81
9. Weaning percentage	89.32 ± 2.52
Carcass performance (N = 25)	
1. Carcass weight (kg)	64.27 ± 0.67
2. Dressing percentage (%)	73.33 ± 0.37
3. Carcass length (cm)	70.62 ± 0.78
4. Back-fat thickness (cm)	2.30 ± 0.21

N, Number of observations

**TABLE 6:** Lifetime production traits of crossbred pig variety (N = 50).

Parameter	Mean ± S.E.
1. Total litter size at birth (no.)	51.83 ± 1.61
2. Average litter size at birth (no.)	9.17 ± 0.17
3. Total litter weight at birth (kg)	44.07 ± 1.29
4. Average litter weight at birth (kg)	7.75 ± 0.14
5. Total litter size at weaning (no.)	47.17 ± 2.69
6. Average litter size at weaning (no.)	8.49 ± 0.20
7. Total litter weight at weaning (kg)	446.19 ± 3.52
8. Average litter weight at weaning (kg)	78.46 ± 1.91

N, number of observations

### 3.4 Experiment III: Performance evaluation of crossbred pigs under a smallholder production system

For performance evaluation of the crossbred pig under a smallholder production system, data were collected from the established 100 units. The crossbred pig performed significantly better than average local pigs under the improved management condition (Table 7). Litter size at birth in the crossbred pigs was 8.87 ± 0.24 and at weaning was 8.27 ± 0.37; they were found to be significantly higher (58–65%) than local pigs under the same management in a smallholder production system. The crossbred pig attained a body weight of 82.54 ± 1.12 kg by 300 days—35–42% higher than local pigs. The number of piglets per sow per year ranged from five to seven in local pigs but 10 to 15 in the crossbred pigs—significantly higher (*p* < 0.01) (Table 7). Hence, the crossbred pigs performed better in terms of both production and reproduction than the local pigs. Incidences of different diseases were recorded: pre-weaning mortality did not differ significantly between crossbred and local average pigs (Supplementary Table S1). However, post-weaning and adult mortality was significantly higher (*p* < 0.05) in crossbred pigs than local pigs under a smallholder pig production system. For market demand between the crossbreed and local pigs based on the survey, the former had a significantly (*p* < 0.05) higher score than that of the latter. However, the score for consumer preference did not differ significantly between the two varieties (Table 7).

**TABLE 7:** Performance of crossbred variety under a smallholder pig production system and its market demand and consumer preference.

Parameter	Average local	Crossbred variety
1. Litter size at birth (no.)	5.83^a^ ± 0.35	8.87^b^ ± 0.24
2. Litter size at weaning (no.)	5.00^a^ ± 0.27	8.27^b^ ± 0.37
3. Body weight at 120 days (kg)	10.85^a^ ± 0.85	24.13^b^ ± 0.56
4. Body weight at 180 days (kg)	18.56^a^ ± 1.05	44.81^b^ ± 0.72
5. Body weight at 240 days (kg)	25.68^a^ ± 0.96	65.31^b^ ± 0.82
6. Body weight at 300 days (kg)	34.17^a^ ± 1.35	82.54^b^ ± 1.12
7. Number of piglets/year/sow	6.23^a^ ± 0.23	13.82^a^ ± 0.38
8. Market demand score	3.67^a^ ± 0.18	4.27^b^ ± 0.20
9. Consumer preference score	4.36^a^ ± 0.24	4.12^a^ ± 0.17

^a-b^ values in the same row with different superscript differ significantly (*p* ≤ 0.05). *Means with the same superscript are not significantly (*p* > 0.05) different in the same rows.

## 4 Discussion

Pigs occupy a unique role among the meat-producing animals of the Eastern Himalayan hill region and are the animal of choice for meat, especially for tribal populations in Northeast India (Talukdar et al., 2019). However, there is a high supply–demand gap in pork due to less-productive pigs under the traditional backyard production system (Mahajan et al., 2015). Crossbred pigs are superior on average than their purebred counterparts under harsh and diverse agro-climatic conditions (Li et al., 2022). Crossbreeding programs take advantage of the effect of individual as well as maternal and paternal heterosis (Versen et al., 2019). To enhance pig productivity in the region, there is an urgent need for the introduction of high-yielding crossbred varieties with indigenous inheritance under the changing climatic conditions. Thus, this study was conducted to develop a crossbred indigenous Niang Megha and Hampshire pig for better adaptability and performance in the hill ecosystem of the Eastern Himalayan hill region of India.

In the first phase, NM was crossed with Hampshire to develop F_1_ (HN-50), HN-75, and HN-87.50; their performance was evaluated to determine the optimum level of exotic inheritance for better adaptability to the region. HN-50 was better in terms of age at sexual maturity, waning percentage, and cost of veterinary medicine than other genetic groups due to higher NM inheritance in HN-50. However, HN-75 was superior to HN-50 for growth performance, litter size at birth and weaning, and for lifetime productivity (Tables 1–3). Based on productive and reproductive performance and disease incidence among the crossbred pigs, those with 75% Hampshire and 25% NM inheritance were selected for crossbreeding development.

Due to the planned crossbreeding program with rigorous selection, crossbred HN-75 pigs attained better adaptability and performance in the hill ecosystem, climatic resilient traits, promising growth rate, and good mothering ability with higher litter size (Banik et al., 2018). However, Kumar et al. (2018) observed that 50% Tamworth × 50% Desi cross pigs (T&D) performed better than 75% Hampshire × 25% Desi pigs due to their higher level of indigenous inheritance. In the present study, after *inter se* mating and selection of HN-75 pigs for six generations, their performance was found to gradually improve, which could be attributable to selective breeding. Production traits were found to have positive and higher genetic gains compared to reproduction traits, since production traits have higher heritability than the latter (Alam et al., 2021). Tribout et al. (2010) also reported similar results for positive genetic gain in production traits on French Large White pigs in two generations of selective breeding. After stability was established for economic traits, the HN-75 pigs were released as “Lumsniang” (*lum* means “hill” and *sniang* means “pig”) (AICRP, 2018).

The crossbred pig was evaluated for its performance and was found to perform well in productive, reproductive, and carcass traits in the hill ecosystem. It attained a body weight of 23.63 ± 0.55 kg at 3 months old and 86.48 ± 0.92 kg at 10 months old. The present finding was comparatively higher than the body weights of indigenous pigs (Niang Megha and Doom) for the corresponding ages (Khargharia et al., 2014), which is due to the Hampshire inheritance. The present findings, however, corroborate the findings of Haldar et al. (2017) who reported that the crossbred pigs—Ghungroo × Hampshire, Tripura Mali × Duroc, and Niang Megha × Hampshire—attained mean body weight of 71.58–89.50 kg at 12 months old. Reproductive performance of the crossbred pig variety was also found to be better than indigenous pigs. However, age at puberty (276.66 ± 2.23 days) and at first farrowing (425.26 ± 2.82 days) of the crossbred pigs in the present study was found to be higher than indigenous pigs like Niang Megha and Doom (Khargharia et al., 2014). The litter size at birth and weaning for the crossbred pigs was 9.02 ± 0.55 and 8.12 ± 0.81, respectively, which is higher than indigenous pigs owing to the Hampshire inheritance. Sharma et al. (2019) reported relatively higher litter size at birth and at weaning in synthetic three-way-cross pigs, Pakhribas in Nepal. Average weight at birth and weaning was found to be 0.92 ± 0.06 kg and 9.46 ± 0.81 kg. Similar findings were recorded in crossbred pigs of Large Black, Saddleback, and Hampshire in Bhutan (Thapa and Timsina, 2018). However, Sharma et al. (2019) reported higher average weight at birth and at weaning in synthetic three-way-cross pigs, Pakhribas in Nepal, than the present study. To study carcass traits, the crossbred pig variety in the present study was slaughtered at 300 days old. The carcass weight was found to be 64.27 ± 0.67 kg and was similar to that of three-way crossbred pigs (25% Large White Yorkshire × 25% Landrace × 50% Duroc) as reported by Sutha et al. (2015); however, they reported lower dressing percentage than the present study's crossbred pigs. The carcass length of these crossbred pigs was 70.62 ± 0.78 cm and back-fat thickness was 2.30 ± 0.21 cm—similar to the reports in crossbred pigs of 50% Tamworth × 50% Desi pigs of Assam by Kalita et al. (2016) and crossbred pigs of 75% Hampshire × 25% NM in Meghalaya (Indian Council of Agricultural Research, 2008). The back-fat thickness of the crossbred pigs in the present study was comparable to the findings of Zhang et al. (2019) in crossbred breeds of China obtained by crossing native Jiaxing Black Pigs with Berkshire, Duroc, and Landrace. Like this study, superior carcass quality was recently recorded in Iberian × Duroc crossbred pigs (Ortiz et al., 2021).

In the present study, the lifetime productivity of the crossbred pigs was calculated for six farrowings. The length of productive life and lifetime production traits are important in commercial swine production because of their association with stability, productivity, and cost of production (Hall et al., 2002). The crossbred pigs in the present study were found to have a total litter size at birth of 51.83 ± 1.61, which correlates well with the findings of Hall et al. (2002) in Meishan crossbred pigs but higher than Duroc crossbred pigs in the United Kingdom. The total litter size at weaning in the present study was found to be similar with that of Duroc crossbred pigs (Hall et al., 2002).

Smallholder production systems are very common in the North-eastern hill region of India, where pigs are reared utilising kitchen swill and free crop residues (Kumaresan et al., 2009). This type of pig production system is economically viable and sustainable at a household level. In the present study, the performance of the crossbred pig variety was compared with that of local average pigs reared under a smallholder production system. The crossbred variety performed significantly (*p* < 0.05) better than the local pigs under the same management conditions. Nath et al. (2013), comparing the performance of local with crossbred pigs under smallholder production system in Sikkim, reported similar findings. The higher incidence of disease in crossbred pigs might be associated with their slow adaptability to the existing environment (Bharati et al., 2022). Litter size at birth of the crossbred variety was 8.87 ± 0.24, whereas at weaning it was 8.27 ± 0.37, which was significantly higher (*p* < 0.05) than the local pigs—results due to the Hampshire inheritance. Haldar et al. (2017) reported similar litter performance in different crossbred pigs in a smallholder pig farming system for Hampshire × Ghungroo, NM × Hampshire, Duroc × Ghungroo, Duroc × Tripura Mali, and Tamworth × Ranchi local pigs. In the present study, the crossbred variety attained a body weight of 82.54 ± 1.12 kg by 300 days under a smallholder production system, which corroborates the findings of Haldar et al. (2017) in NM × Hampshire crossbreds in smallholder farms. Hence, rearing the crossbred pig variety under a smallholder production system was better in terms of production and reproduction performance and resulted in more profitability than average local pigs under the same management system.

## 5 Conclusion

The Lumsniang crossbred pig variety performed better in terms of productive and reproductive traits, besides having better adaptability in the hill ecosystem, over the existing indigenous/average pigs in the Eastern Himalayan hill region. Furthermore, the crossbred pigs performed better than local pigs under a low-input traditional production system. Large-scale dissemination of the crossbred variety in the smallholder production system is possible by introducing nuclear breeding farms at a district level in collaboration with state departments. Large-scale propagation of the crossbred pig variety could lead to increases in production, productivity, livelihood, and income of the region's farmers.

## Data Availability

The raw data supporting the conclusion of this article will be made available by the authors, without undue reservation.
