# Supplementary material for: Performance of crossbred pigs with indigenous and Hampshire inheritance under a smallholder production system in the Eastern Himalayan hill region
Source: Front Genet. 2023 Apr 3;14:1042554. doi: 10.3389/fgene.2023.1042554 (PMC10106676; doi:10.3389/fgene.2023.1042554)
Supplement: Supplementary file 1 [file Table1.DOCX]

| **Supplementary Table. 1. Incidence (%) of major disease conditions under smallholder pig production system** | | |
| --- | --- | --- |
| **Parameters** | **Nondescript** | **Crossbred variety** |
| **A. Pre-weaning Mortality (%)** | 8.24^a^±1.21 | 8.63^a^±0.91 |
| i) Weak piglets | 0.06^a^±0.00 | 0.08^a^±0.00 |
| ii) Crushing | 0.62^a^±0.03 | 1.10^b^±0.16 |
| iii) Piglets Diarrhoea | 6.16^a^±1.01 | 6.27^a^±0.96 |
| iv) Other causes | 1.41^a^±0.31 | 1.32^a^±0.52 |
| **B. Post weaning Mortality (%)** | 2.38^a^±0.61 | 3.42^b^±0.82 |
| i) Piglet Diarrhea | 1.23^a^±0.61 | 2.01^b^±0.61 |
| ii) Pneumonia | 0.56^a^±0.01 | 0.53^a^±0.03 |
| iii) Wound/abscess /ear bit/leg and hoof lesion/other body lesions/ | 0.57^a^±0.00 | 0.91^b^±0.11 |
| **C. Adult mortality (%)** | 0.67^a^±0.10 | 1.31^b^±0.12 |
| *Means with the same superscript are not significantly (P<0.05) different in the same rows | | |
